# 

**Published:** 1867-01

**Authors:** 


### CHICAGO

### MEDICAL JOURNAL.

VOL. XXIV. JANUARY, 1867. NO. 1.

CHICAGO:

GEORGE H. FERGUS, BOOK AND JOB PRINTER,

12 & 14 CLARK STREET.

1867.

